# A Tailored Web-Based Video Intervention (ParentCoach) to Support Parents With Children With Sleeping Problems: User-Centered Design Approach

**DOI:** 10.2196/33416

**Published:** 2022-04-19

**Authors:** Katharina Preuhs, Hilde van Keulen, Rosa Andree, Sophie Wins, Pepijn van Empelen

**Affiliations:** 1 Expertise Group Child Health Netherlands Organization for Applied Scientific Research (TNO) Leiden Netherlands

**Keywords:** positive parenting, usability testing, lower health literacy, user-centered design, iterative development, eHealth, web-based intervention, mobile health, mHealth, parenting

## Abstract

**Background:**

Many parents frequently struggle with undesirable or problematic behavior (ie, temper tantrums and whining) displayed by their child. To support parents in promoting positive parenting skills (ie, recognizing challenging situations and reacting appropriately), the interactive video e-learning tool *ParentCoach* was developed. The tool aims to teach parents generic behavioral responses by means of situational learning, tailoring, and problem solving. The first demonstration focused on sleeping problems.

**Objective:**

The aim of this paper is to illustrate the user-centered development of ParentCoach.

**Methods:**

We conducted usability, understandability, and acceptance tests among the target group (29 parents, 7 youth health care professionals, and 4 individuals with former lower health literacy) in different phases of the development process via focus groups, interviews, and surveys. This allowed for relevant insights on specifications and user requirements to guide the development and revision of the tool in each iteration.

**Results:**

Iterative testing and development allowed for the final demonstration of ParentCoach to be experienced as a relevant and accessible parenting intervention that can be used as a stand-alone program or in combination with another program.

**Conclusions:**

This paper elaborates on the iterative development process and its benefits for the final demonstration of ParentCoach.

## Introduction

### Background

Research has shown that 36% of parents with children living at home report concern regarding the upbringing of their children. Among this group, 60% look for help and advice outside their family, friends, and social environment [1]. In addition, an evaluation among users of the parental information platform GroeidGids (Growth Guide) in the Netherlands, a platform with >100,000 users yearly, showed that parents often have questions regarding diet, sleep, health, and parenting [2]. Hence, there is a substantial group of parents who do not know how to deal with common problems in their children, such as anxiety, stubbornness, disobedience, or temper tantrums, which may emerge in a certain developmental stage of the child [3]. It is important to provide early parenting education, advice, and support based on scientific knowledge about healthy and adaptive parenting. Effective parenting may prevent aggravation of relatively small and manageable problems, which could otherwise result in more severe behavioral problems [4]. This might especially be true for vulnerable families (ie, lower-educated, lower-income, or migrant families) as these groups are more likely to experience problems that can negatively influence their parenting style (eg, less consistent or supportive parenting) [5].

The upbringing of children may affect various aspects early on, including social skills, brain development, self-control, emotional regulation, mental and physical health, and resilience [6]. According to Sandler [6], “effective parenting” consists of adequate discipline practices, advice, and guidance and a positive affective relationship between the parent and the child as well as instances to improve children’s skills to adjust to environmental demands. A systematic meta-analysis by Kaminski et al [7] revealed several parenting strategies to be especially effective, such as consistently responding to one’s child and using “time out” as a disciplinary strategy, with active acquisition of parenting skills revealing significantly large effects. In addition, improving parenting practices has been shown to benefit the well-being and overall development of both parents and children [8].

In the Netherlands, there are a number of effective parenting interventions available, but most focus on secondary or tertiary prevention; that is, the promotion of parenting skills for parents at high risk who already experience serious problems [9]. Moreover, most of these interventions are time-consuming; they consist of multiple individual or group meetings. This creates logistical difficulties for parents, such as inconvenience regarding location, transport challenges, limited flexibility in work schedules, and lack of childcare [10]. In addition, parents perceive stigma or concerns about confidentiality, which affects their willingness to participate in these interventions [10]. These interventions may also result in low program recruitment and retention as they require significant training- and supervision-related time and costs [11,12]. This poses a significant burden on participants and professionals, which potentially undermines their reach and impact [12]. The number of effective primary preventive parenting interventions is sparse [13]. There is a clear need for more easily accessible and effective primary preventive interventions that go beyond information provision [12,14].

Technology-assisted primary preventive parenting programs may be effective in overcoming some of the barriers of parenting programs. In a recent meta-analysis, the effectiveness of technology-assisted parenting interventions was examined, assessing the effects of 9 different programs among disadvantaged families [10], including programs such as Triple P Online [15], Parent-Child Interactions-Cellular [16], and Infant-Net [17]. This meta-analysis showed that such interventions can be effective in supporting positive parenting behavior and have a positive impact on parental well-being and child behavior. However, this study revealed considerable variation between programs. The difference between programs seemed related to programs that focused solely on information and demonstration of parenting principles and programs that also provided tailored feedback and advice (generally via phone or email). Programs without tailored advice and feedback proved far less effective than programs with tailored advice and feedback.

In this study, we describe the development of *ParentCoach*, a low-demand program that can be used depending on the experienced need of parents and to overcome potential taboos or reluctance to take part in parenting programs. Importantly, ParentCoach addresses some of the omissions of previous technology-assisted programs by not only providing information and demonstration of parenting practices but also providing a web-based environment to practice with common difficult parenting situations, in which parents receive personalized feedback and advice. Hence, we aim to make use of the advantages of technological possibilities and ensure tailored advice, which has been shown to improve the effectiveness of previous parenting programs, with the ultimate goal of enabling parents to practice adequate parenting skills to prevent more generally experienced parenting problems.

### ParentCoach

ParentCoach is a web-based, tailored, interactive video e-learning tool [18]. A demonstration version of ParentCoach was developed to promote positive parenting skills with regard to children’s healthy sleeping behaviors. This demonstration version provides a practice context with exercises and advice content, including tips and an explanation of how to apply the evidence-based principles. As sleep is a common concern for parents, ParentCoach was first developed with sleep in mind. The practice content addresses different aspects of getting children to bed by means of situated learning (ie, what to do when your child does not want to go to bed) and repeated rehearsal of (coping) skills (ie, how to cope with temper tantrums). Tips and resolutions are delivered through an animated female character as a relational agent.

ParentCoach consists of three scenarios in which parents can practice their parenting skills regarding the sleeping behaviors of their child (ie, *it’s bedtime*, *putting on the pajama*, and *going to bed*). These scenarios depict a common situation that parents may experience to be difficult (ie, the child does not want to put on her pajama). Following short sequences in which a short dialogue between parent and child is shown, parents can choose between 2 options on how to react. In response to their choice, a relational agent provides instant feedback and reinforcement and offers further explanation of why a certain choice was (less) adequate. In each scenario, the child may act in different ways (ie, obedient, refusing, whining, or angry). Although the situation may be the same, as the child reacts differently, parents are expected to adapt their response (ie, reinforcing positive behavior or adequately responding to negative behavior). Thus, 12 practice situations can be completed. An overview of the scenarios can be found in the *Results* section. In these scenarios, parents can practice or are advised on how to deal with negative child behavior. In addition, they can receive advice on reinforcing positive child behavior. Therefore, parents can choose to receive a total of 6 tips regarding positive and negative behavior (eg, *reward your child*, *teach your child new things*, or *temper tantrums* and *refusal*). Upon opening the link, parents can decide whether to watch a short introduction or another segment. The intervention can be performed in one’s own time and repeated as many times as necessary as there are no time-bound restrictions. ParentCoach does not provide any additional human support as users interact with the animated web-based coach who provides tips and reacts to the users’ choices on a 24/7 basis. Thus, the tool can be seen as an extension of other parenting programs.

ParentCoach was iteratively developed based on user-centered design principles, which means that the end user was closely involved in the developmental process to fine-tune the intervention as much as possible to their needs and preferences. Such a user-centered design approach is important as it enhances usability and user engagement and, hence, increases the likelihood of an intervention being effective and feasible [19,20]. Its multiple iterative cycles of concept generation, prototype design, and evaluation reveal important needs and preferences of the target group and result in enhanced usability and acceptability of the design by the end users [19,21]. Furthermore, early-stage prototype testing in terms of usability prevents the aggravation of use-related issues in later stages of the developmental process [19]. On the basis of early user acceptance testing, certain features are still flexible and can be further adapted or disregarded before a substantial amount of time, money, and effort has been invested without matching the users’ requirements [22]. eHealth interventions often do not fit people with lower literacy skills and do not involve them in the intervention development [23]. This study also involved people with low literacy skills in the iterative user-centered design to examine and optimize the fit of the ParentCoach intervention to their needs and preferences.

In the following sections, we describe the phase-wise development and iterative testing of ParentCoach based on sleep problems in children.

## Methods

### Overview

ParentCoach was iteratively developed based on user-centered design principles. This process consisted of the following phases: (1) definition phase, (2) concept testing, (3) prototype testing, (4) usability testing, and (5) low-literacy testing. Potential end users (parents) and intermediate users (youth health care professionals [YHCPs]) were involved in the process by means of user-based assessments. An advisory board of professionals and parents provided feedback during the developmental process. First, the behavioral problem and program objectives were defined. Evidence-based methods were translated into behavioral strategies and included in ParentCoach. In the second phase, a concept version of ParentCoach was developed and tested among YHCPs in a focus group interview (n=3). The third phase consisted of incorporating the received feedback into the prototype of ParentCoach and testing this prototype among parents (n=3) and YHCPs in individual interviews (n=4). In the fourth phase, ParentCoach was tested among parents by means of a web-based survey (n=26). Finally, ParentCoach was evaluated among (former) people with low literacy skills (n=4) to ensure its acceptability and usability among vulnerable families. For each interview, a protocol was developed. All interviews (individual or group) were recorded. The input from the interviews was assessed using thematic analysis based on a topic list. The topic list consisted of topics such as relevance, usability, layout, and acceptability of the prototype intervention and was based upon the interview protocol. For each phase, a summary was made of the interviews by means of the topic list. The data from the web-based survey (phase 4) were processed using SPSS (version 25; IBM Corp), and descriptive statistics (frequencies, means, and SDs) were used to analyze the results. An overview of the different steps and sample characteristics can be found in Table 1.

**Table 1:** Overview of the iterative development steps and sample characteristics.

Step	Goal	Means	Sample
Definition phase	Definition of the behavioral problem and program objectives, translation of evidence-based methods into behavioral strategies	Literature study	N/A^a^
Concept testing	Storyboard development testing and testing of a concept version of ParentCoach: perceived relevance and user requirements	Focus group	3 female YHCPs,^b^ mean age 38.7 (SD 4.5) years
Prototype testing	Early prototype development testing: exploring the acceptance and relevance of the script dialogue decision tree underlying ParentCoach	Individual interviews	3 mothers, mean age 34.3 (SD 2.1) years, and 4 female YHCPs, mean age 39 (SD 11.3) years
Usability testing	Refinement and usability (and acceptance) testing among the target group	Web-based survey	26 mothers, mean age 30.4 (SD 3.15) years
Low-literacy testing	Ensuring acceptability and usability of ParentCoach among vulnerable families	Individual interviews	4 parents with (former) low literacy skills

^a^N/A: not applicable.

^b^YHCP: youth health care professional.

To ensure the anonymity and privacy of the participants, data were processed in an anonymized way. Personal information was not included in the reports of the interviews and surveys, and the contact information of the participants was deleted after they received a reward for participation.

### Ethics Approval

ParentCoach was approved by the ethical board of the Netherlands Organisation for Applied Scientific Research (Institutional Review Board, 2018-068).

## Results

### Definition Phase

Sleep disturbances are strikingly common among preschool-aged and school-aged children (aged 2-6 years), with between 20% and 42% of these children experiencing sleep problems [24]. Sleep problems in children include bedtime resistance, night awakenings, short sleep duration, daytime sleepiness, and problems with falling asleep. These problems have been associated with behavioral, emotional, learning, memory, and health problems among children [25-28]. Sleep problems among young children also negatively affect parents and families; that is, parents may experience stress, sleep deprivation, health problems (including chronic fatigue), maternal depression, and irritability, whereas problems among families can be expressed as poor parenting strategies, family dissatisfaction, and relationship problems [29,30]. Thus, supporting parents to prevent sleeping problems among children is crucial for the health of both children and families.

Although it is essential for parents to learn how to deal with such problems, in the Netherlands, there are no proven effective programs to prevent sleeping problems among young children [31,32]. Providing preventive information alone is not sufficient; it has been shown that effective parenting is characterized by the ability to cope with difficult situations by implementing specific child interaction strategies. Moreover, repeated provision of problem situations in which parents can role-play and engage in situational learning activities (eg, calming, strategic ignoring, and consistent behavior) may enhance the effectiveness thereof [7,33]. Parents become more effective in interacting with their child or children when they are trained in positive behaviors (eg, enthusiasm, interest, and doing things together), are responsive to the emotional and psychological needs of their child, use time-outs efficiently, react to their child consistently, and learn which of their behaviors negatively and positively influence child (sleeping) behavior [6,7,34]. Situated learning and repeated rehearsal of (coping) skills are evidence-based methods to train parents in positive child interactions [7] and may help parents avoid child sleeping problems. Therefore, we formulated the following program objectives: to enable parents to (1) recognize challenging situations and (2) adequately react by training them in generic actions as suggested by the literature using evidence-based techniques, called *positive parenting principles*.

First, key elements and behavioral techniques were selected to match the goals of ParentCoach.

To realize the program objectives, ParentCoach will apply behavioral methods such as situational learning, tailoring, and problem solving. Possible applications identified were communication strategies such as narratives (also storytelling), video feedback in recognizable situations, or audiovisual materials to accomplish teaching generic behavioral options.

Situational learning of effective parenting practices could be stimulated by guiding a user through different scenarios in which the interaction between a parent and a child is observed so that they can interact, for example, by choosing an adequate reaction. Narrative communication has been shown to contribute to better recognition, relevance, realism, and active learning [35] and may increase active processing and understanding of the content [36]. In addition, narratives have been shown to improve comprehensibility in individuals with varying levels of health literacy [37] and, therefore, will be applied. Moreover, the use of audiovisual information (as opposed to textual information) can improve attention and comprehension in groups with lower socioeconomic status (SES) [38,39].

To improve information processing, active involvement, enhanced personal relevance, and positive effects on skills compared with universal interventions [40-42], tailoring could be an effective method to provide personalized feedback and scenarios depending on the user’s choice. Tailoring (health) messages enhances the attractiveness, salience, and, thereby, effectiveness of the information [43], which may consequently lead to greater maintained behavior changes [44].

To facilitate the attainment of problem-solving skills, users may practice with a variety of potential problem scenarios that also integrate a range of different dialogues (eg, the child immediately listens to the parent vs a child who is reacting in a whiny manner). The application of various situations enhances the learning experience by providing the user with a greater number of practice situations as well as personalized feedback, which reinforces the learned content so that parents can practice different parenting strategies.

On the basis of the objectives and chosen methods stated above, a first concept was developed, which was consecutively tested in 4 iterative phases to be discussed in the following sections.

### Storyboard Development and Testing

To test the idea of ParentCoach, a storyboard was developed. This test aimed to examine perceived relevance and user requirements. The concept scenario was tested by means of a focus group interview with 3 YHCPs in December 2017. Professionals were recruited via the child health center in Leiderdorp, the Netherlands. The interviewees were all female (3/3, 100%) with a mean age of 38.7 (SD 4.5) years and 10.5 (SD 6.1) years of work experience mostly with children aged between 0 and ≥7 years.

**Figure 1:**
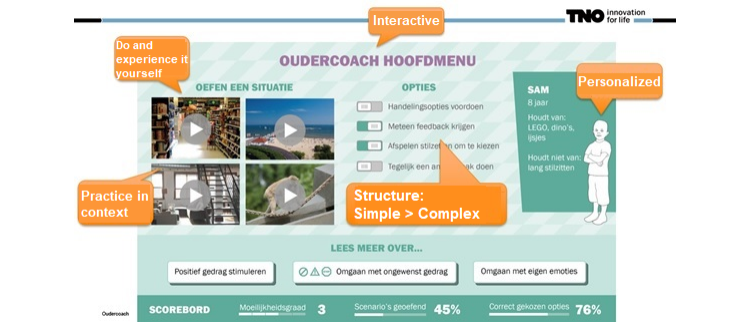
Mock-up of the main menu of ParentCoach.

**Figure 2:**
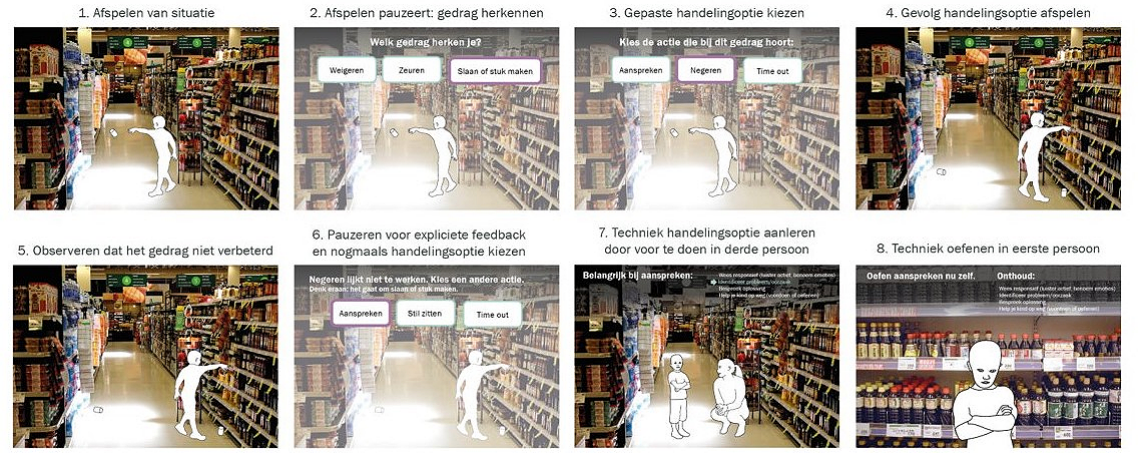
The scenario as depicted in the first iteration.

The first concept of ParentCoach incorporated a scripted 2D-based storyboard describing possible intervention features and functionalities. The storyboard as presented to the YHCPs offered various contexts (eg, at the beach or zoo) where parents could practice parenting skills in different situations that varied in degree of difficulty. In addition to personalization of the settings, including when to receive feedback and whether to suggest options on how to act, users were offered the possibility to personalize the child. A scoreboard sought to indicate the number of situations the user had already engaged in as well as a *score* of correct answers given (Figure 1). A particular scenario was created (*supermarket scene*): while grocery shopping, the child is shown displaying rebellious behavior (throwing something; Figure 2). The user is then provided with a menu of options to indicate what kind of behavior they recognize in the situation (eg, refusal or whining), followed by the choice to react in a certain way (such as ignoring it or taking a time-out). Upon choosing a reaction, the scenario displays how the child reacts to the user’s response.

Regarding added value, all YHCPs (3/3, 100%) indicated that the scenario of ParentCoach was useful. The envisioned program was assessed to be “fun” in terms of enjoyment, and the mode of delivery was said to be innovative by all interviewees (3/3, 100%). Furthermore, the appearance was rated as attractive. Approximately 67% (2/3) of the health professionals reported that they would advise ParentCoach to all parents, whereas 33% (1/3) stated that they would rather advise the program to parents with parenting problems. The YHCPs found ParentCoach to be relevant, but certain points of improvement were shared. Although approving of the overall script and feedback provided to the user, the professionals indicated the concept to be relevant and realistic.

Hence, the evaluation strengthened the idea of the potential utility of ParentCoach. In addition, the YHCPs made improvement suggestions for further development of ParentCoach. Specifically, they pointed out that we should ensure that accessibility and the low literacy of potential users were taken into account, such as the use of simple language and starting off with the basic principles with potential for expansion on the topic.

### Early Prototype Development and Testing

**Figure 3:**
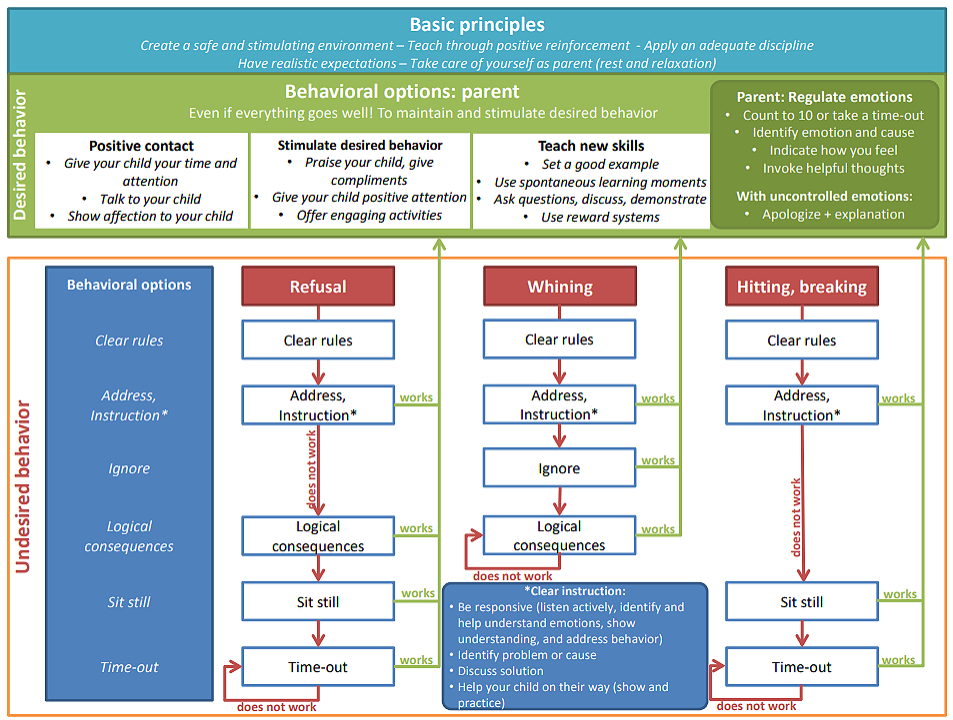
Basic positive parenting principles underlying ParentCoach.

Next, a prototype was developed. First, a logical sequence of actions was defined (Figure 3).

This model provides the user with instructions based on an operationalized problem. This consists of a stepwise instruction on how to (1) reinforce desired behavior and (2) discourage the undesirable behavior of a child. The scheme aims to offer users a structured approach to positive parenting and can be downloaded by the users.

Actions were then translated into scripts to mimic a dialogue between parent and child.

First, for the interactive videos, dialogues were scripted, and decision trees were defined to be able to tailor videos. The decision tree includes (1) type of situation, (2) type of child response, (3) type of options parents could choose, and (4) feedback from the relational agent, resulting in 24 potential situations. An example of such a decision is presented in Table 2.

The scenarios were integrated into an interactive video prototype and tested in the second iteration. This test explored the acceptance and relevance of the script dialogue decision tree underlying ParentCoach.

The updated prototype was assessed among 3 parents and 4 YHCPs by means of individual interviews in October 2018. Parents were recruited via flyers at the child health center in Leiderdorp, the Netherlands; via an advertisement at Kidsproof (a regional site for parents of young children with activities in the region of the Hague, the Netherlands); and via an email to parents of young children working at the Netherlands Organisation for Applied Scientific Research, Leiden, the Netherlands. All parents were female (3/3, 100%) with a mean age of 34.3 (SD 2.1) years. Their highest completed level of education was higher education, and they had 1 or 2 children aged between 2 and 5 years. YHCPs were recruited via the child health center in Leiderdorp, the Netherlands; via the Municipal Health Service in Leiden, the Netherlands; and via an email to a national network of YHCPs. All YHCPs were female (4/4, 100%) with a mean age of 39 (SD 11.3) years, had between 7 and 22 years of work experience, and worked with children in all age groups. This test focused, among other things, on attractiveness, utility, identification, and recognition. Questions elaborated, for example, on users’ first impression of the program, the added value of the program according to the user, in which cases the user would want to use ParentCoach, and how they experienced the overall visualization.

**Table 2:** Extract from the decision tree indicating the possible dialogue between the user and coach in the case of the child (1) listening to the mother instantly and (2) showing refusal behavior within the scenario *It’s bedtime*.

Type of situation and possible scenarios			Child response		Parent response options					Feedback from ParentCoach		
					Parent+		Parent–		Parent+			Parent–
“**It’s 6:45, bedtime. I want you to put on your pajama.”**												
	Child listens	“Okay I will put on my pajama. Be right back.”		“Great. How nice that you do this so quickly. I’ll be right there.”		“Hurry up hey.”		Well done. This was positive behavior. In this case, you can indeed react with a compliment.			Look out. Your child shows positive behavior. Reward this; for example, by giving a compliment.	
	Child refuses	Child goes on playing		“I want you to put on your pajama.”		“Okay, 5 more minutes then.”		Well done, your child shows refusal behavior. Clear instructions are required here!			Look out. Your child displays refusal behavior. Do not reward this, but stick to your rules.	

In general, the evaluation indicated that the examples were useful, and the scenarios were recognizable, short, and accessible. The relational agent was said to be pleasant. However, the options of parenting reactions that users could choose were declared to be too obvious, with the user experiencing a tendency toward the “right choice.” This implied that the user did not have the full opportunity to learn from their mistakes as they were not provided with information on why a certain choice may be less adequate. Furthermore, concerning visualization, the mother in the scenario was said to be illustrated too much like a grandmother, and the explanation given by the coach was said to be too long and difficult to understand.

The professionals evaluated ParentCoach as relevant, especially for “short, simple problems.” However, they suggested potential adaptations regarding the inclusion of lower-SES populations as well as taking cultural factors into account without stereotyping. Another suggestion to increase relevance comprised the provision of an explanation concerning the importance of the positive parenting principles.

Mothers experienced the scenarios in ParentCoach as recognizable. The child’s voice and way of talking were said to be very realistic. Concerning the use of the program, the interviewees experienced ParentCoach as a short (ie, not too time-consuming), accessible program that could be used in one’s own time. Both the fact that the coach was an independent character and the delivery format of ParentCoach as a whole were strong points according to the mothers. However, the amount and length of information and tips given by the coach were said to be too much as well as too scientific. The interviewees reported that they preferred alternations between explanations, short example movies, and exercises.

Both parties (parents and professionals) indicated a felt need to be able to get in contact with one another in case there was an indication of the need to provide or receive professional human support, which suggests embedding ParentCoach in the broader context of youth health care services (YHCSs). On the basis of the results of this test round, ParentCoach was further adapted taking into account the feedback received.

### Refinement and Usability Testing

The third iteration comprised a survey evaluating usability and acceptance among the target group. This version incorporated adaptations and improvements based on the feedback and suggestions retrieved during the second iteration.

The participants evaluated the 3 animation figures that were shown in the videos (ie, the mother, child, and coach), the assignments and skill training, the design, and the idea of ParentCoach. Thereby, we assessed the third version of the web-based ParentCoach, which was complemented with a web-based survey among a convenience sample of 26 participants. Recruitment took place in March 2019 via Facebook advertisements targeting young mothers with children aged 2 to 6 years. The participants were female parents, each with 1 to 3 young children in the age range of 2 to 5 years. The mean age of the participants was 30.4 (SD 3.15) years. Most participants were higher educated (21/26, 81%) and had a Dutch background (23/26, 88%).

The survey consisted of close-ended and open-ended questions. Close-ended questions related, among other things, to the videos; for example, *I experienced practicing with the videos to be (i) certainly clear, (ii) certainly not educational, (iii) certainly not recognizable*, to be answered on a 7-point Likert scale, or *How did you experience the mother character?* regarding (1) recognizability, (2) trustworthiness, or (3) irritation, again to be answered on a 7-point Likert scale. Furthermore, the questions concerned the explanations given by the coach (eg, *I experienced the explanations of the coach to be (i) relevant, (ii) clear, (iii) educational, (iv) easy to follow*, to be answered on a 7-point Likert scale). Open-ended questions assessed what the users experienced as most and least pleasurable, what they missed, and tips and recommendations for further development.

Of the 26 participants, 24 (92%) reported having watched the practice videos, whereas 23 (88%) watched the explanation videos (referring to tips and basic parenting principles). Positive points taken from this usability test revealed that the situations were recognizable, and the overall program was accessible, concise, and clear. The relational agent was evaluated most positively (7.3 out of 10) of the animation figures, followed by the mother (7.0) and the child (6.9). In general, 46% (12/26) of the users reported that they would use ParentCoach themselves, whereas 81% (21/26) would recommend it to someone else. Table 3 shows the overall usability and acceptability scores of ParentCoach. The most positive ratings (all scores ≥4 on a 5-point scale) were for (1) ease of use, (2) accessibility, and (3) recognizable situations. Nevertheless, *fit for personal purpose* was rated lower (3 on a 5-point scale).

**Table 3:** Usability and acceptability of ParentCoach (N=26).^a^

Item	Rating, mean (SD)
ParentCoach illustrates situations that I personally recognize.	4.12 (1.18)
ParentCoach teaches me what to do in challenging parenting situations.	3.62 (0.75)
ParentCoach includes practical tips.	3.77 (0.71)
ParentCoach fits my needs well.	3.08 (1.16)
ParentCoach stimulates me to apply the exercises and explanations at home.	3.50 (1.07)
ParentCoach is a helpful means to practice.	3.69 (1.19)
ParentCoach is accessible.	4.35 (0.69)
ParentCoach is easy to use.	4.31 (0.68)
ParentCoach is fun to use.	3.85 (0.97)

^a^Likert scale (range 1-5).

From the open-ended questions, we could gather recommendations for improvement. The participants perceived the child as somewhat unrealistic in difficult situations. The feedback and explanation by the coach were found to be too wordy and complicated. In addition, users missed the possibility to either pause or replay certain segments. Some participants pointed out that the assignments were too short and should consist of more information about how to react in a certain situation (eg, when your child does not want to go to bed). Parents also reported that they wanted to receive more emotional support (eg, through the coach saying that practicing can be hard and that there are moments when parenting strategies may not work even though they are doing their best). Finally, parents requested more options, further indicating that the coach should emphasize that, besides the given parenting principles, other parenting strategies could also be good.

### Low-Literacy Testing

On the basis of the last iteration, further improvements were made. A final evaluation was aimed at also assessing a fit with people with lower health literacy skills given that the convenience sample did particularly comprise people with higher education. This final prototype was evaluated with 4 participants who had (formerly) low literacy skills by means of individual interviews in May 2019. Participants were recruited from a test panel of language ambassadors of ABC Foundation in the Netherlands, a panel consisting of members with (formerly) low literacy skills. All participants (4/4, 100%) had children; 50% (2/4) had young children, and the other 50% (2/4) had adult children. This test focused on evaluating ParentCoach in terms of accessibility for users with low literacy, poor health skills, and low abstract thinking ability. Low-literacy testing consisted of methods requesting the testers to read aloud written text (eg, *Would you please read aloud what is written here?*), summarize and teach back information in their own words (eg, *What did you just hear?* *What do they mean with* refusal behavior*?* or *What can you do here?*), and give advice on how to rephrase certain expressions to increase comprehensibility (eg, *What was (un)clear?* *How would you resolve this?*).

The test panel was very positive and enthusiastic about ParentCoach and its features. The testers perceived the content as useful and comprehensible, and the videos were perceived as instructive. For this reason, they graded ParentCoach with an 8.3 on a scale of 1 to 10. The panel also mentioned some points of improvement. For instance, they pointed out that a short introduction video should be added to explain how ParentCoach works and how to use it, with an explanation of the menu. Furthermore, to enhance usability and acceptability, the panel advised the adaptation of some specific design features (eg, increasing font size and adapting symbols and text to enhance readability) and syntax (eg, rewording *undesirable behavior* into *negative behavior* and explaining difficult words). Reported difficulties included the time indication integrated in the menu. Here, users mistook the length of the video sequence (0:20) as the time when the action should take place (ie, 20 minutes after midnight). To resolve this issue, the indication was clearly labeled *duration*. In addition, examples of options for action were asked to be illustrated through animation. All feedback and advice from the test panel was processed in the final version of ParentCoach.

### Finalization of Prototype

**Figure 4:**
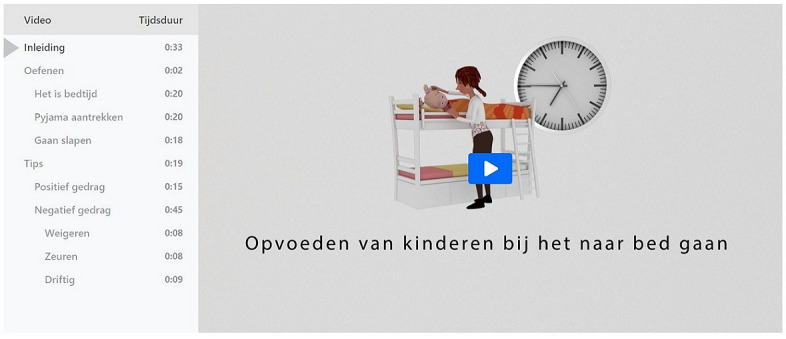
Introductory home screen of ParentCoach (on the left hand side, the menu of the interactive video is shown).

On the basis of the input from the last evaluation, a final version of ParentCoach was created [14]. As a result of user-centered design and iterative testing, an introductory movie was included explaining how ParentCoach can be used. The menu included enables the user to either run chronologically through the program or choose from 3 practice scenarios or advice regarding positive and negative behavior (Figure 4). In each of the 3 scenarios, the audiovisual material is delivered within 18 to 20 seconds. Scenarios to practice positive parenting principles include *it’s bedtime*, *putting on one’s pajama,* and *going to bed*. The scenarios start with a short sequence in which the problematic behavior of the child is depicted (eg, the mother makes it clear that it is time to go to bed, and the child whiningly responds saying that she does not want to). Following the short sequence, parents are offered two reactions; for instance, *I want you to put on your pajama. It is quarter to seven* or *Okay, 5 more minutes then*. Upon choosing one of the possible reactions, the user receives immediate feedback from the animated coach, providing the user with advice and information regarding the given response. To enable users to practice with different situations, there are several ways in which the child may react so that the user is offered a broad range of situations to foster their learned skills.

In addition to practicing skills through scenarios, ParentCoach provides several tips and advice (eg, having positive contact or rewarding the child). Parents can either choose between positive or negative behavior as a response to the dialogue with the coach or directly select the desired element through the menu. Each piece of advice includes certain examples of how to stimulate positive behavior (eg, *give your child a compliment* by praising how well the child tidied up their room by themselves). This advice is accompanied by animations that support the audio text. Advice regarding negative behavior includes the topics *refusing*, *whining*, and *fierce behavior*, which can be accessed via the dialogue with the coach or in the menu. These behaviors are addressed in the enlisted steps that the user can take to engage with a child displaying the respective behavior. Each step is followed by a consecutive step in case the earlier measure failed to succeed.

## Discussion

### Principal Findings

In this paper, we elaborated on the application of a user-centered approach toward the development of the innovative web-based video tool ParentCoach. The tool empowers parents to recognize challenging situations and teaches them generic behavioral responses to stimulate the positive behavior of their child or children by means of interactive video e-learning using behavioral techniques such as situational learning, tailoring, and problem solving.

As a result of iterative usability testing, adaptations were made to ParentCoach to address wishes and needs and overcome perceived obstacles. These included the addition, adaptation, or extraction of some features such as text blocks, certain ambiguous symbols, optical change of the coach character, and look of the virtual environment. In addition, the spoken text was changed in terms of (1) ease of wording and (2) breaking up texts into smaller chunks. Furthermore, the response options were adapted content-wise as well as regarding syntax for easier understanding. To make ParentCoach more educational, response options were altered to ensure that the most appropriate response was not too obvious, thereby contributing to a better learning environment for parents. Low-literacy testing enabled the program to be inclusive.

ParentCoach may provide relevant evidence-based tips and practice situations that empower parents to tackle everyday parenting challenges without the need for further human support, thereby relieving pressure on the health care system and increasing the efficiency of support provision. Moreover, the basic parenting principles processed in ParentCoach comply with the national sleep guidelines for preventive child health care professionals [45]. Overall, ParentCoach was well received by parents and professionals. Parents reported (1) recognizable situations; (2) useful examples; (3) accessible use; (4) a pleasant coach; and (5) understandable and clear text, audiotext, and videos. Approximately 46% (12/26) of participants indicated wanting to use ParentCoach, whereas 81% (21/26) reported that they would recommend the tool.

In summary, ParentCoach offers a unique, low-demand, and accessible tailor-made preventive parenting support program in which we hope to have overcome some of the limitations of previous technology-enabled programs [10] that did not provide a practice setting with tailored feedback and advice. ParentCoach could be used as a stand-alone program or be integrated into other parenting programs or YHCSs such as the web-based platform Growth Guide, a platform by YHCSs. Its features allow users flexibility regarding time and duration to pass through the scenarios and tips as suggested by the literature advocating addressing such limiting factors and barriers that comprise childcare and transportation issues [46,47]. By means of ParentCoach, parents are enabled to train their parenting skills in their own time in the comfort of their own home. As such, it seems an important contribution to the existing information-only or secondary preventive interventions, as was recognized by the YHCPs in the study and a recent review by the steering committee of Growth Guide (representing 18 YHCSs); it also addresses commonly experienced problems of a considerable number of parents in the general population. Furthermore, ParentCoach is unique in its approach as it is built to be inclusive for all parents and, therefore, seems particularly useful for parents with lower health literacy skills as it combines tailoring, interactive learning, and the use of both audio and visual information—strategies that have been shown to be effective in improving the attention and comprehension of this particular group [38,39]. Inclusive design in eHealth applications ensures that products are accessible and usable by the majority and, hence, helps overcome health inequalities, especially for lower-SES groups as it further minimizes the risk of growing health inequalities [48]. Therefore, cocreation with lower-SES groups is a means to ensure that ParentCoach is accessible and functional for everyone.

As pointed out by Davis and Venkatesh [22], early acceptability testing among the target group offers various benefits in the development process. First, initial acceptability testing allows for the prevention of potential pitfalls early on as it provides relevant insights into barriers and postimplementation user acceptance. Thus, it is crucial to promote user acceptability testing early in the design process as it may mitigate later financial deficits because of failing to meet the users’ needs and wishes for functionalities before making significant investments [22]. As the expected user population may fall short, predicting user acceptance may not only save significant losses but also guide important decisions in the design process. On the basis of usability testing, certain initial decisions may be abandoned or progressed in an alternate way to focus on certain functionalities to match the end users’ needs, as was done during the development of this program. Early testing goes along with a low cost compared with the implications of a huge investment that is not accepted and used by the end user [22].

### Conclusions

As a conclusion, early testing of relevance and understanding of factors that contribute to esthetics, utility, understanding, and usability enabled the progressive development of ParentCoach according to the needs and wishes of the potential end users. Cyclical testing among this group positively affected the overall interface and interaction by revealing and correcting certain barriers. Moreover, the user-centered development of ParentCoach in cocreation with participants with lower health literacy skills contributes to the literature in that it offers an example of inclusive development.

Although in this paper we have shown that the working principle of ParentCoach can work, future research may address the extent to which the program actually enables parents to learn and apply positive parenting principles. Furthermore, future directions may include the incorporation of different contexts and other common problems that parents face, such as eating behavior, screen time, or physical activity.

## Abbreviations

SES: socioeconomic status
YHCP: youth health care professional
YHCS: youth health care service
